# Isolated Jejunal Perforation Following Bicycle Handlebar Injury in Adults: A Case Report

**DOI:** 10.1155/2013/678678

**Published:** 2013-08-05

**Authors:** Kyriakos Neofytou, Maria Michailidou, Athanasios Petrou, Sakis Loizou, Charalampos Andreou, Marios Pedonomou

**Affiliations:** Department of Surgery, Nicosia General Hospital, Palaios Dromos Lefkosias-Lemesou, No. 215, Strovolos, 2029 Nicosia, Cyprus

## Abstract

The small intestine is the third in frequency intraperitoneal organ which is injured after blunt trauma of the abdomen. In most of the cases, this type of injuries is accompanied by other injuries, which make it more difficult to diagnose. Failure of diagnosis and delay in treating these injuries significantly increase the morbidity and mortality of these patients. Abdominal visceral injuries after flipping the handlebar of the bike are common in children. Such injuries can cause injury to both solid and hollow abdominal viscera. Unlike children, adults' abdominal visceral injuries after flipping the bike's handlebar are extremely rare. A 25-year-old man was admitted to our department due to progressively abdominal pain after an accident with the handlebar of his bike. The subsequent CT scan after per os administration of contrast medium revealed the presence of free intraperitoneal contrast. It is a rare case of jejunal perforation after flipping the handlebar of the bicycle which was treated by partial removal of the injured part of jejunum and end-to-end anastomosis. To the best of our knowledge this is the first time we describe such an injury with this mechanism to an adult.

## 1. Introduction

Intra abdominal organ injuries caused by a stroke of bike handlebars are well documented in children. Flipping the bicycle's handlebars in the abdomen is common in children and can cause a variety of injuries such as traumatic rupture of solid organ (liver, spleen, pancreas, and kidney), rupture of hollow organs (intestine, stomach), posttraumatic hernias of the abdominal wall, and even injuries of intra-abdominal vessels (ruptured abdominal aorta, traumatic arterial occlusion) [1–7]. Apart from significant morbidity in children, these injuries are combined with a huge financial cost to health systems and beyond [8].

The abdomen is the third most common anatomical area of the body which undergoes blunt injuries, and 75% of these injuries are due to road-traffic accidents [9]. Although the lesions of the small intestine follow the frequency of liver and spleen injuries after blunt abdominal trauma, such frequency is small and limited to less than 5% of total injury [10]. The injuries are in most cases accompanied by other ones, which increases both the mortality of the patients and the difficulty in dealing with them [11].

Isolated lesions of the jejunum secondary to blunt trauma of the abdomen are very rare injuries, and few reports exist in the literature in the form of case reports [12–14].

Injuries from the bicycle's handlebar are very rare in adults, and an indication is the absence of literature on such injuries. This fact can be attributed both to differences in behavior between adults and children (much less use of bicycles by adults, more careful use than children) and anatomical differences. The main difference is that the most powerful abdominal wall of adult partly protects intra abdominal viscera from such injuries.

Here, we present a rare case of an adult with isolated rupture of jejunum after injury by the bicycle's handle bar.

## 2. Case Report

A 25-year-old man was admitted to the emergency department after falling off his bicycle. He was hit by the bicycle's handle bar into the left side of the abdomen. The clinical examination revealed a fit young man who had only a scratch (abrasion) on the abdominal wall left of the umbilicus. Hemodynamically he was normal and alert and referred to intense pain at the abrasion site. The abdomen was soft, without signs of peritoneal irritation while sensitivity was only at the point of the abrasion. Laboratory tests, including white blood cells, liver enzymes, and amylase, were normal. The abdominal X-ray in upright (standing) position did not reveal the presence of free intraperitoneal air. Abdominal ultrasound did not reveal any solid abdominal organ injury nor the presence of free intraperitoneal fluid. The pain was attributed to hematoma of the rectus abdominis muscle pricey below the abrasion which was revealed by ultrasound.

**Figure 1:**
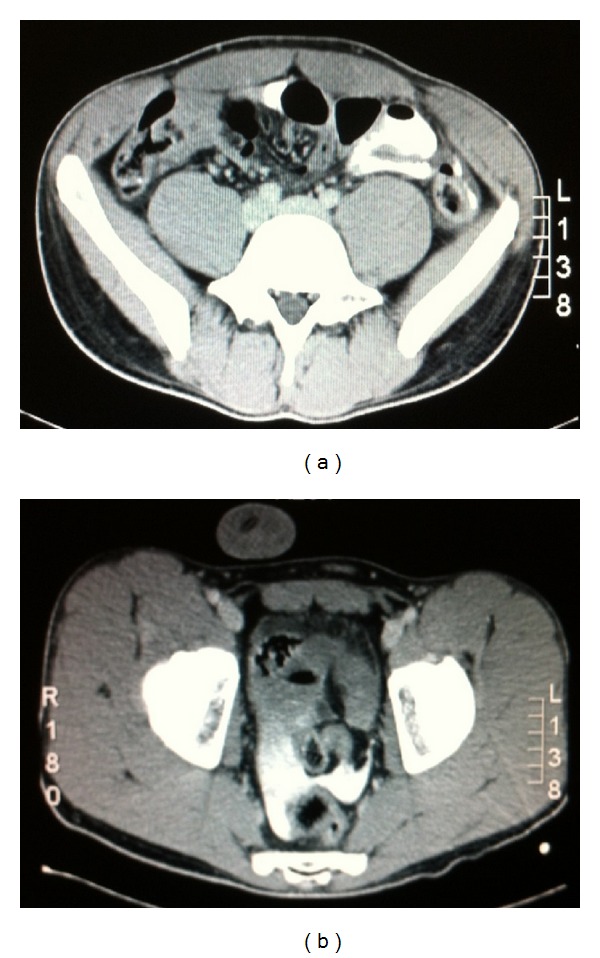
(a) Free intraperitoneal contrast in left paracolic groove. (b) Significant amount of free contrast in the pelvis.

The pain of the young man was gradually worsening and was mostly described diffusely throughout the whole abdomen. The abdomen remained soft and tender without signs of peritoneal irritation. We proceeded to the administration of per os contrast material and CT abdomen. The CT scan revealed the presence of sufficient amount of free intraperitoneal contrast but did not allow the clarification of the exact point of escape. The free contrast material was detected at the left side of the abdomen, at the area of the descending colon but mainly in the pelvis (Figure 1). The intraluminal contrast had even reached up the level of the transverse colon.

**Figure 2:**
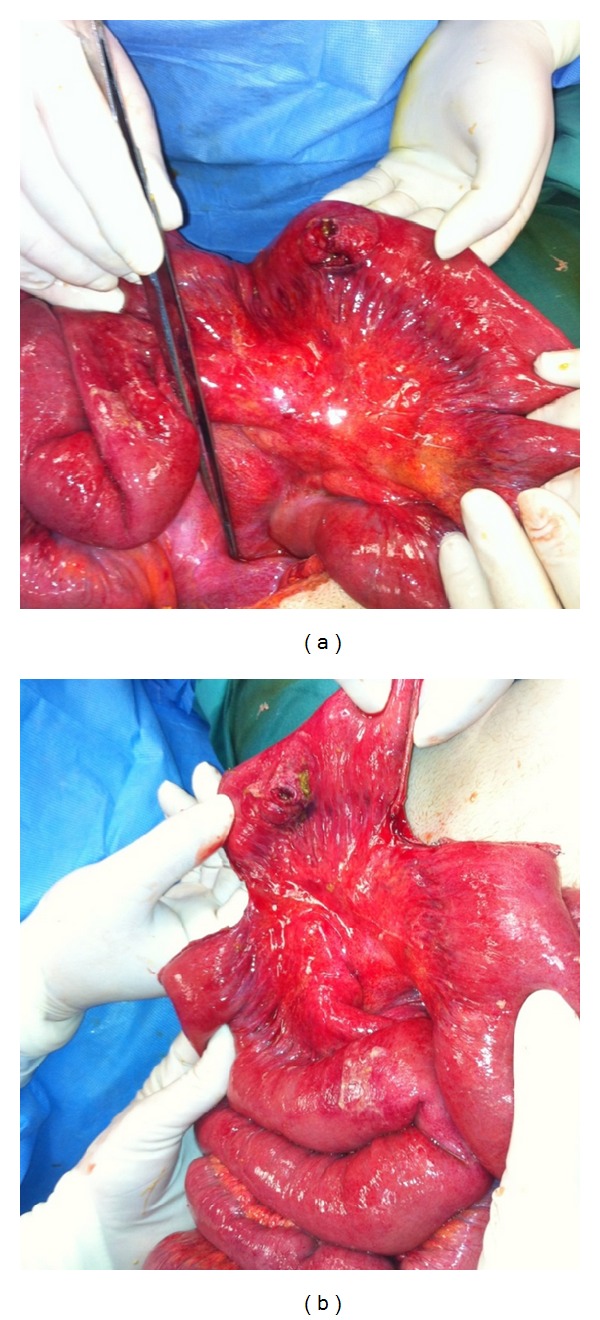
(a) Traumatic rupture of the jejunum. Extensive lesion of the serous layer at the site of the rupture. We can see the ligament of Treitz. (b) Partial removal of jejunum.

The patient was taken to the operating room for exploratory laparotomy immediately after the CT scan (4 hours after the arrival at the emergency department and about 4.5 hours after the time of injury). Intraoperatively, fecal peritonitis due to isolated rupture of jejunum 30 cm from the ligament of Treitz was revealed. The rupture was at the center of an extensive jejunum serous layer fissure giving the picture of “blow out” perforation. At the jejunal mesentery there was minimal bruising. Multiple washings of the peritoneal cavity were performed with normal saline, and the restoration of jejunal injury was done with partial resection and end-to-end anastomosis (Figure 2).

The postoperative course of the patient was uncomplicated, and he was discharged on the 6th postoperative day. 9 days later he returned with clinical and radiological signs of ileus that led us to the operating room 48 hours later due to deterioration of his clinical picture. The cause of the ileum was adhesions, so we proceeded to lysis of adhesions. The anastomosis was normal without leakage. Five days later the patient was discharged, and a year later he is still free of symptoms.

## 3. Discussion

The lesions of the small intestine and even if it is the third in frequency site of injury after blunt abdominal trauma do not exceed 5% of all abdominal injuries [10]. Although the main cause of blunt abdominal injuries is road traffic accidents [9], in children one of the mechanisms of blunt injury of the abdomen is flipping the bicycle's handlebar [8]. Flipping the handle bar in the abdomen in children may cause injury of all abdominal organs including the small intestine [1–7].

The energy transferred to the injury site in the abdominal wall from the handlebar due to its small diameter acts as a javelin releasing the energy of injury to a small area of the abdominal wall. This, in combination with the relatively weak abdominal wall in children, puts the underlying viscera of the abdominal wall in danger. Both the stronger abdominal wall of adults and the less numerous strokes by bicycle make this mechanism of injury to the abdominal organs rare in adults.

In our patient, this transfer of energy through the abdominal wall led to pressure on the initial part of jejunum. The extensive serous lesion in the vicinity of the jejunum rupture leads to the conclusion that probably at the time of injury this part of the small intestine was full of content, and the transfer of energy from the injury resulted in a significant increase of the intraluminal pressure leading to a “burst” of the bowel.

The diagnosis of lesions of the small intestine after blunt trauma is often particularly difficult as the signs and symptoms of peritonitis appear in a later time. The clinical examination is not reliable in the diagnosis of these lesions as the classical triad of rupture of the small intestine (hard abdomen, sensitivity, and lack of intestinal sounds) is present only in 30% of patients with rupture of the small intestine [10]. The presence of free intraperitoneal air on abdominal X-ray after traumatic rupture of the small intestine is the exception rather than the rule [15].

Our patient was not the exception to the rule as both the initial clinical examination and abdominal X-ray in a standing position gave no signs of a possible ruptured hollow organ. The clinical examination is particularly useful and sensitive diagnostic test if repeated in small periods of time because the patient will develop signs of irritation of the peritoneum progressively.

For the diagnosis of rupture of the small intestine, the diagnostic peritoneal lavage (DPL) and the focused abdominal sonography for trauma (FAST) have been used [16].

Nowadays, the investigation of choice for early diagnosis of lesions of the small intestine is a CT scan with contrast administration per os. As for the clinical examination, so as for the FAST, the sensitivity of the method is increased if there is enough time given for free intraperitoneal fluid to be concentrated in sufficient quantity so as to be detected with FAST. The CT shows sensitivity and specificity of 92% and 94%, respectively, in the evaluation of the patient with blunt abdominal trauma, and most importantly it displays negative predictive accuracy of 100% which enables us to exclude early any possible lesions of the small intestine [17] and thus contributes to better treatment of those patients [18]. The main findings of CT diagnosis leading to rupture of the small intestine are the presence of free intraperitoneal fluid or contrast allocated per os, the thickening of the wall of the small intestine, and of course the presence of free intraperitoneal air. Notably intraperitoneal free air occurs in less than 50% of the cases [19].

The main disadvantage of CT is that, for its execution, the patient has to be hemodynamically stable. The general condition of the young man in our case gave us the option to move to CT, and the examination was performed approximately two hours after per os administration of contrast. Although it was not possible to pinpoint the source of leak of contrast from the gastrointestinal tract, the recognition of the presence of free intraperitoneal contrast was an absolute indication to proceed to exploratory laparotomy directly. Free intraperitoneal air was not observed.

Although there is no consensus in the literature regarding the effects of delay in treatment of traumatic rupture of the small intestine, the widely accepted position is that the sooner treated, the lower the morbidity for the patient [20]. Failure of early diagnosis and treatment of such lesions is one of the main causes of increased mortality in blunt trauma, in which additionally the diagnosis becomes more difficult as both clinical and even many of the radiological signs of rupture of the small intestine may be coated by accompanying injuries [21].

## 4. Conclusion

Both the rarity of rupture of the small intestine and the inherent difficulties in diagnosis of these kinds of injuries make the need of the physician to have a high index of suspicion imperative, based on the mechanism of injury. The presentation of the above case aims to raise awareness of doctors regarding the possibility of rupture of the small intestine in adults following abdominal injury with the bicycle's handlebar. Knowledge of this rare mechanism of injury takes on added value as more and more adults use bicycles.
